# [Retracted] Regulatory effect of microRNA-223-3p on breast cancer cell processes via the Hippo/Yap signaling pathway

**DOI:** 10.3892/ol.2026.15874

**Published:** 2026-09-21

**Authors:** Tonghua Du, Dan Wang, Xiaoyu Wan, Jingwei Xu, Qi Xiao, Bin Liu

Oncol Lett 22: 516, 2021; DOI: 10.3892/ol.2021.12777

Following the publication of the above paper, a concerned reader drew to the Editor's attention that, regarding the Transwell invasion assay data shown in Fig. 4B on p. 6, the ‘Control’ and ‘NC’ data panels were overlapping, such that data which were intended to show the results of differently performed experiments had apparently been derived from the same original source. In addition, the data panels showing the results of scratch-wound assay experiments (for the 24 h time point) in Fig. 4A contained apparent anomalies, namely the repeated patternings of small, but identifiable areas of cellular data within the same panels.

After having conducted this independent investigation of the data in the Editorial Office, the Editor of Oncology Letters has determined that this paper should be retracted from the Journal on account of a lack of confidence in the scientific integrity of the data. The authors were asked for an explanation to account for these concerns, but the Editorial Office did not receive a satisfactory reply. The Editor regrets any inconvenience that has been caused to the readership of the Journal.

